# COVID-19 outbreak among employees of a German hospital: risk factor analysis based on a follow-up questionnaire and seroprevalence

**DOI:** 10.1007/s15010-024-02220-1

**Published:** 2024-03-15

**Authors:** Jennifer Kosenkow, Juliane Ankert, Michael Baier, Miriam Kesselmeier, Mathias W. Pletz

**Affiliations:** 1 Institute for Infectious Diseases and Infection Control and Center for Sepsis Care and Control (CSCC), Jena University Hospital/Friedrich-Schiller-University, Am Klinikum 1, 07747 Jena, Germany; 2 Institute of Medical Microbiology, Jena University Hospital/Friedrich-Schiller-University, Jena, Germany; 3 Institute of Medical Statistics, Computer and Data Sciences, Jena University Hospital/Friedrich-Schiller-University, Jena, Germany

**Keywords:** COVID-19, Health care worker, Nosocomial transmission, Outbreak report, SARS-CoV-2, Seroepidemiologic studies

## Abstract

**Background:**

The Co-FriSero study describes a COVID-19 outbreak at the Friedrichroda hospital in Thuringia, Germany, with 185 beds and 404 employees, at the onset of the pandemic between March 30th, 2020, and April 13th, 2020. This study aimed to analyze potential sources of SARS-CoV-2 transmission amongst hospital employees.

**Methods:**

After the outbreak, a comprehensive follow-up was conducted through a questionnaire and a seroprevalence study using two different immunoassays for IgG detection and a third for discordant results.

**Results:**

PCR screenings confirmed SARS-CoV-2 infection in 25 of 229 employees, with an additional 7 detected through serology. Statistical analysis indicated that direct patient contact, exposure to high flow ventilation in non-isolated rooms, direct contact with colleagues, shared use of recreational rooms, and carpooling were associated with an increased infection risk. Conversely, contact with family and friends, public transportation, public events, and use of locker rooms were not associated with infection. Male gender showed a lower infection likelihood, independent of age and other risk factors.

**Conclusion:**

This study highlights the role of direct patient care and internal staff interactions in the spread of SARS-CoV-2 in the hospital setting. It suggests that non-traditional transmission routes like carpooling require consideration in pandemic preparedness.

## Introduction

In December 2019, a pneumonia cluster emerged in Wuhan, attributed to a novel beta coronavirus (SARS-CoV-2) by January 2020 [5, 9, 10]. This highly transmissible virus spreads through respiratory droplets, close contact, and contaminated surfaces [14]. By March 2020, the outbreak was declared a pandemic [1, 18]. In Germany, the first case was recorded on January 28th 2020, and had, by October 11th 2023, 38.5 million cases and 176,200 deaths, while globally there were about 696 million cases with 6.9 million deaths (Statista Research Department, 2023). The severity of COVID-19 ranges from infections with mild respiratory symptoms to severe systemic complications [2, 4, 7, 16]. Within this study, conducted between October 19th 2020 and November 18th 2020—when vaccines were not yet available—we investigated a regional hospital outbreak, with a focus on seroprevalence, protective measure efficacy, and risk stratification. The protocol was based on a prior study from the Jena University Hospital and aimed at detailing the outbreak causes, protective measure effectiveness, and SARS-CoV-2 antibody prevalence among 229 participating staff members (i.e., 56.6% of the eligible staff members).

## Methods

### Study design and setting

The Co-FriSero study (“Investigation of the outbreak of COVID-19 infection at the community- hospital Waltershausen-Friedrichroda and survey of seroprevalence and infection status of SARS-CoV-2 among facility staff”) is an outbreak report with a follow-up cross-sectional cohort study. This study was conducted between October 19th 2020 and November 18th 2020 at the community hospital Waltershausen-Friedrichroda in Germany. Friedrichroda is a small town with 7115 inhabitants in the district of Gotha in the state of Thuringia, which is located in the center of Germany. The hospital with 185 beds has a regional care mission and 404 employees (as of August 16th 2021).

Research was conducted in accordance with the Declaration of Helsinki and national and institutional standards. The study was approved by the ethical committee of the Thuringian Chamber of Physicians (approval no. 2020–1873). This outbreak was reported following the ORION guideline (https://www.ucl.ac.uk/amr/Reporting_Guidelines/ORION) for outbreak reporting.

### Enrolment and definition of exposure risk

Inclusion criteria were that the subject was an employee of the hospital, 18 years of age or older, and willing to sign a written informed consent form. According to their profession (e.g., physician, nursing staff, reception area and administrative staff) and workplace (e.g., intensive care unit, emergency department, normal ward, administration), the employees were classified to predefined risk areas (low, medium, high) indicating the risk of a contact with a COVID-19-positive patient. Occupational groups outside these areas, such as laboratory staff who had contact with COVID-19-positive specimens but no patient contact, staff who were not available during the scheduled study period and the backup appointment, and staff who did not provide a blood sample, were excluded. The low-risk group included administrative and kitchen staff who had no patient contact. The medium-risk group included staff who were in contact with patients but did not routinely work with confirmed or suspected COVID-19 patients (e.g., operating room staff, functional areas, normal wards). The high-risk group included hospital staff who routinely cared for confirmed or suspected COVID-19 patients (e.g., intensive care unit, intermediate care unit, emergency department, isolation ward).

### Questionnaire

Each participant was asked to fill a questionnaire. The questionnaire included questions on age, occupation, education level, exposure to COVID-19-positive patients, results of previous polymerase chain reaction (PCR) tests, and clinical symptoms such as sore throat, headache, cough, loss of taste and smell, and diarrhea. The questionnaire also included questions to assess the risk of nosocomial transmission asking about personal contact with confirmed COVID-19 patients or their environment, about compliance with hygienic measures and wearing personal protective equipment (PPE). We surveyed the symptoms and hygiene measures retrospectively, but in relation to the time of the outbreak. We also asked about symptoms, compliance with hygiene guidelines and possible exposure risks during the outbreak period. These questions were based on the WHO “Assessment of risk factors for coronavirus disease 2019 (COVID-19) in health workers: protocol for a case–control study” (https://www.who.int/publications/i/item/assessment-of-risk-factors-for-coronavirus-disease-2019-(covid-19)-in-health-workers-protocol-for-a-case-control-study).

### SARS-CoV-2 antibody testing

Primary testing for the presence of SARS-CoV-2 antibodies was performed using two different IgG detection immunoassays: chemiluminescence immunoassay technology (CLIA) LIAISON (DiaSorin) and enzyme immunoassay EDI Novel Coronavirus SARS-CoV-2 IgG ELISA (Epitope Diagnostics Inc.). Both assays target the presence of antibodies to recombinant nucleoside capsid proteins and were performed according to the manufacturer's instructions. With two matched positive test results in both immunoassays, the participant was classified as SARS-CoV-2-seropositive and with two concordant negative test results, the participant was classified as seronegative. In the event of discordant results, a third test (recomeLine SARS-CoV-2 IgG immunoassay, microgene) was used according to the manufacturer’s instructions to make a final decision. In this test, the diagnostic markers, nucleocapsid protein and spike protein are set as immunodominant antigens. For all three tests, both sensitivity and specificity were reported by the manufacturer to be high (> 98%).

### Statistical methods

Characteristics of study participants (overall and stratified by SARS-CoV-2 test result) were summarized as both absolute and relative frequencies, as well as median with first and third quartiles (Q1, Q3). Logistic regression modelling according to Firth was used to investigate the association between seropositivity of study participants working in different COVID-19 risk areas and potential risk factors as well as clinical symptoms. In univariable models, we chose SARS-CoV-2 seropositivity (binary) as the dependent variable and the respective factors under investigation as independent variables. Additionally, we adjusted these models for age and gender to account for these possible confounders. To receive comparable results between an univariable and the respective multivariable model, we excluded participants without information on age and gender from the univariable models. We report (adjusted) odds ratios (OR) together with their 95% confidence intervals (CI) and *p*-value. Logistic regression modelling was performed with R (version 4.2.2) and the R package logistf (version 1.25.0). All remaining analyses were done with SPSS Statistics version 27.0 for Apple (IBM Corp., Armonk, NY, USA).

## Results

### SARS-CoV-2 outbreak at the hospital

#### Infection control measures before the outbreak

At the beginning of the pandemic (January 2020), there was regular information from the hospital administration on the infection control measures recommended by the Robert Koch Institute (RKI) and its implementation monitored by the hygiene specialist and hygiene officers. In March 2020, a task force consisting of management, medical director, hospital epidemiologist, infection control practitioners and other was established within the hospital. Recommendations for the daily work routine were created by this board. A leaflet on hygiene measures regarding SARS-CoV-2 was prepared, regularly updated according to RKI guidelines and made available to all employees. The elective surgery program was reduced to the most necessary operations, visitors were only allowed in special situations, cleaning intervals were increased (especially those of the door handles), employees were trained to handle and care for COVID-19 patients, especially in the intermediate care unit (IMC), intensive care unit (ICU) and emergency room with appropriate protective equipment. A ward was converted to an isolation ward, and checklists for the care of COVID-19 patients were designed. All of this was done in advance, before the first SARS-CoV-2-positive patient was admitted as an inpatient on March 23rd 2020.

#### Outbreak description

**Fig. 1:**
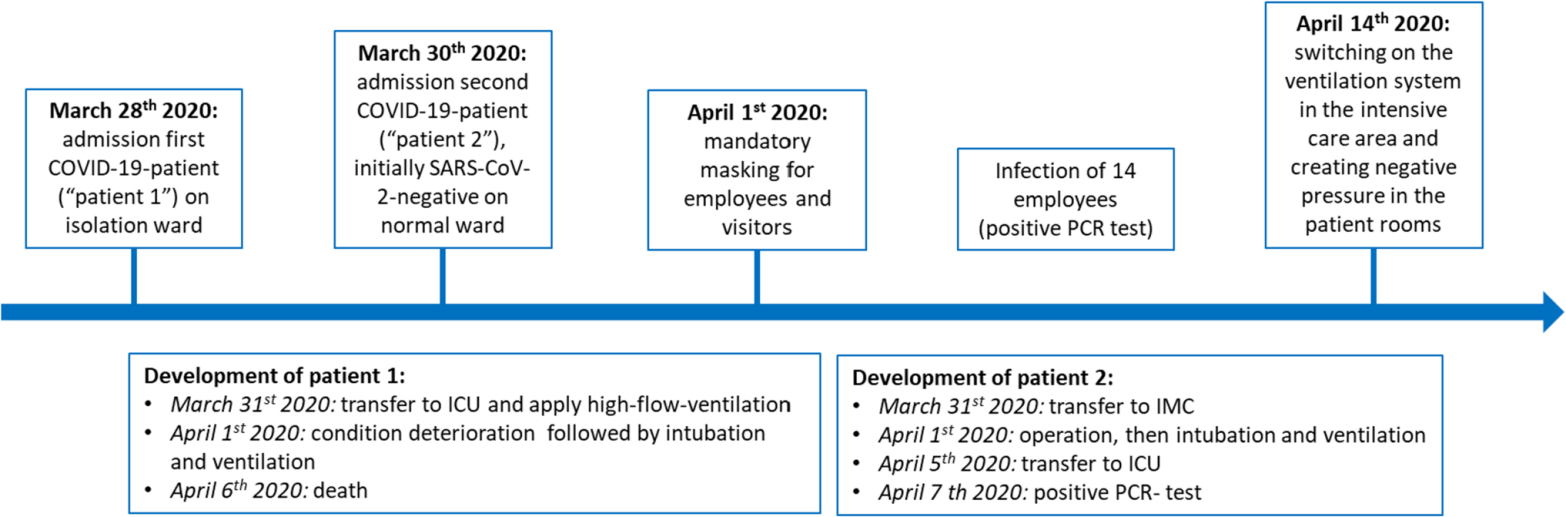
Timeline of events in the beginning of the outbreak at the hospital. This figure includes an overview of the first COVID-19 patients, as well as their further progression within the hospital. *ICU* intensive care unit, *IMC* intermediate care, *PCR* polymerase chain reaction

The outbreak among the health care workers occurred in the period between March 30th 2020 and April 13th 2020. During this time, 3 patients and 25 of 229 workers with PCR-confirmed SARS-CoV-2 infection were identified. An overview of the course (including the first two SARS-CoV-2-positive patients) is provided in Fig. 1.

The first patient, who was tested positive for SARS-CoV-2, was tested positive on admission and admitted to the isolation ward on March 28th 2020. Due to detoriation, the patient was transferred to the ICU on March 31st 2020. Here, he was provided with high-flow ventilation. On April 1st 2020, he had to be intubated and ventilated. During this period, 14 staff members, both physicians and nurses-all wearing a surgical mask, became infected and were tested positive for SARS-CoV-2 by PCR. The patient died on April 6th 2020 as a result of his COVID-19 infection.

The second patient tested positive for SARS-CoV-2 was tested negative on admission and admitted to a regular ward on March 30th 2020. He was transferred to an IMC unit on March 31st 2020, underwent surgery on April 1st 2020, was still being ventilated transferred to the ICU post-surgery and extubated on April 5th 2020. Two days later, he was tested positive for SARS-CoV-2.

The third patient tested positive for SARS-CoV-2 had already been admitted to the hospital on March 24th 2020 due to an underlying disease and was tested negative for SARS-CoV-2 on admission. During his hospitalization, the patient spent some days (from April 1st to 6th 2020) in the IMC unit. Because of a suspected COVID-19 contact, the patient was subsequently transferred to the isolation ward because COVID-19 contact was suspected. As the patient's condition worsened, he was again transferred to the IMC unit on April 8th 2020 and treated by high-flow therapy. A PCR test on April 9th 2020 revealed a SARS-CoV-2 infection. The patient died on April 12th 2020.

#### Outbreak response

Regular audits with the health department and an external hospital epidemiologist were implemented. Regular testing of hospital employees with rapid antigen tests was introduced, and employees were also instructed to fill out a Corona diary, including questions about symptoms and their regularly measured body temperature. All employees who tested positive by PCR were followed up by regular testing and were not allowed to return to work until a negative PCR test result was obtained and symptoms had completely resolved. Patients with suggestive symptoms were tested by a rapid SARS-CoV-2 antigen test during admission. The initially inactivated ICU ventilation system was activated and set for negative pressure on April 14th 2020.

### Description of the risk groups

**Fig. 2:**
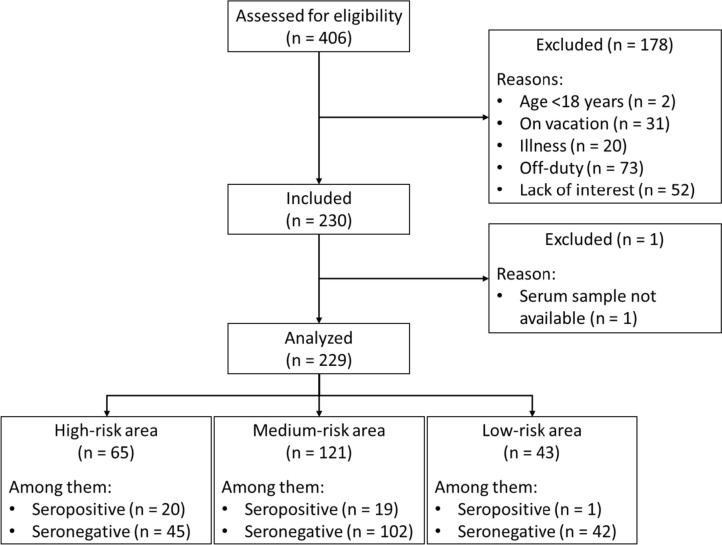
Flowchart of the Co-FriSero study. The number of study participants (n), the reasons for exclusion from the study, and the classification of hospital employees into the 3 categories according to the risk of coming into contact with SARS-CoV-2-infected patients is presented

We included 229 (56.7%) participants in the study who met our inclusion criteria (see Fig. 2). Among them, 201 (87.7%) were female and 28 (12.3%) were male. The median age of the participants was 47 (Q1-Q3: 35–55) years. The most common occupational group included nurses (*n* = 98, 43.2%), followed by administrative staff (*n* = 22, 9.7%) and physicians (*n* = 21, 9.3%). In our study cohort, 65 (28.3%) individuals were classified within the high-risk category, with 20 of them exhibiting seropositivity in SARS-CoV-2 IgG antibody testing. The medium-risk group, comprising 121 (52.8%) individuals, contained 19 (47.5%) individuals tested seropositive. The low-risk category encompassed 43 (18.8%) individuals, among whom only 1 (2.3%) individual exhibited seropositivity. Within the high-risk group, 56.9% (37 out of 65) of staff members reported direct contact with confirmed COVID-19 patients. Among these individuals, 4 had received specific training for managing COVID-19 cases, while 10 were uncertain about their training status. Furthermore, 16.9% (11 out of 65) of employees in the high-risk group acknowledged private contact with individuals infected with SARS-CoV-2 in their community. In contrast, 10 employees in the medium-risk group had encounters with COVID-19 cases beyond the hospital, whereas only 1 individual in the low-risk group reported contact with SARS-CoV-2 positive individuals outside the hospital environment. Further characteristics of the cohort are provided in Table 1.

**Table 1:** Characteristics of the study participants—overall and stratified by the result of a SARS-CoV-2 antibody test (positive/negative)

Characteristic	Overall (*N* = 229)	SARS-CoV-2 serological status	
		Positive (*N* = 40)	Negative (*N* = 189)
Demographics			
Age, in years^*1^	47.0 (35.0; 55.0)	45.5 (32.5; 55.2)	47.0 (37.0; 55.0)
Male gender^*2^	28 (12.3%)	9 (22.5%)	19 (10.2%)
COVID-19 risk group (according to work area)			
High risk	65 (28.4%)	20 (50.0%)	45 (23.8%)
Medium risk	121 (52.8%)	19 (47.5%)	102 (54.0%)
Low risk	43 (18.8%)	1 (2.5%)	42 (22.2%)
Profession*^2^			
Doctor	21 (9.3%)	5 (12.5%)	16 (8.6%)
Nurses	98 (43.2%)	30 (75.0%)	68 (36.4%)
Physician assistant	10 (4.4%)	1 (2.5%)	9 (4.8%)
MTA/RTA	12 (5.3%)	0 (0.0%)	12 (6.4%)
Among them:			
MTA	5 (2.2%)	0 (0.0%)	5 (2.7%)
RTA	7 (3.1%)	0 (0.0%)	7 (3.7%)
Operating room nurse/anesthesia nurse	19 (8.4%)	0 (0.0%)	19 (10.2%)
Among them:			
Operating room nurse	13 (5.7%)	0 (0.0%)	13 (7.0%)
Anesthesia nurse	6 (2.6%)	0 (0.0%)	6 (3.2%)
Physiotherapist	10 (4.4%)	1 (2.5%)	9 (4.8%)
Cleaning Staff	7 (3.1%)	0 (0.0%)	7 (3.7%)
Technology			
Administration	22 (9.7%)	1 (2.5%)	21 (11.2%)
Other	28 (12.3%)	2 (5.0%)	26 (13.9%)
Potential source of infection			
Contact to infected colleague	10 (4.4%)	8 (20.0%)	2 (1.1%)
Changing room	1 (0.4%)	1 (2.5%)	0 (0.0%)
Recreation room	4 (1.7%)	4 (10.0%)	0 (0.0%)
Carpool	2 (0.9%)	2 (5.0%)	0 (0.0%)
Failure to maintain the minimum distance	3 (1.3%)	2 (5.0%)	1 (0.5%)
Non-compliance with hygienic measures	2 (0.9%)	1 (2.5%)	1 (0.5%)
Direct contact to infected patient	16 (7.0%)	15 (37.5%)	1 (0.5%)
Nursing activity on infected patient	13 (5.7%)	12 (30.0%)	1 (0.5%)
Highflow ventilation	6 (2.6%)	6 (15.0%)	0 (0.0%)
Family/Friends	3 (1.3%)	2 (5.0%)	1 (0.5%)
Public transport	1 (0.4%)	0 (0.0%)	1 (0.5%)
Public events	2 (0.9%)	0 (0.0%)	2 (1.1%)
Summary sources of infection			
Professional contact^*3^	12 (5.2%)	8 (20.0%)	4 (2.1%)
Private contact^*4^	4 (1.7%)	2 (5.0%)	2 (1.1%)
Patient-related contact*^5^	22 (9.6%)	20 (50.0%)	2 (1.1%)

MTA, medical technical assistant; N, number of; RTA, radiological technical assistant

^*1^Information missing for 4 individuals who did not test positive

^*2^Information is missing for 2 individuals who do not have a positive test result

^*3^Colleagues, changing room, recreation room, carpool, failure to maintain the minimum distance

^*4^Family/Friends, public transport, public events

^*5^Patient, nursing activities, highflow ventilation

Characteristic

^*3^Colleagues, changing room, recreation room, carpool, failure to maintain the minimum distance

^*4^Family/Friends, public transport, public events

^*5^Patient, nursing activities, highflow ventilation

### Seroprevalence and reported symptoms

We detected antibodies in 40 out of 229 (17.5%) employees. Thirteen (5.6%) employees who had a negative result in in one of the two primary tests (CLIA or ELISA) were retested by microgene. Here, ten employees showed a seropositive result. To test accuracy, 35 samples with negative CLIA and ELISA results were also tested by the microgene test. All of them exhibited negative results.

**Fig. 3:**
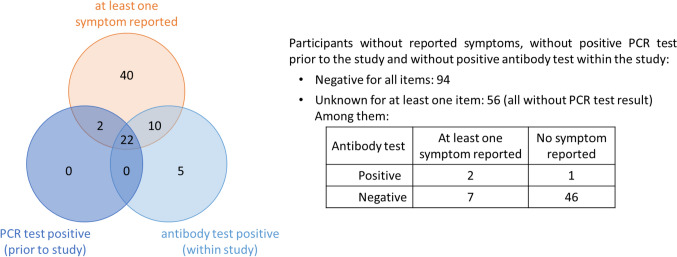
Venn-diagram displaying the overlap between seropostivity, positive PCR test and a reported symptomatic course. Information in participants with missing information are provided next to the diagram. *PCR* polymerase chain reaction

Among the 229 participants, 173 reported about at least one PCR test results prior to the study. Among them, 24 reported at least one positive PCR test prior to the study. All of them reported at least one symptom and 22 of these PCR-positive subjects were also tested seropositive within the study. Further 12 seropositive participants reported symptoms, while 6 reported no symptoms. Forty-seven participants reported symptoms, but were seronegative within the study and provided no positive PCR test results prior to the study. All infected employees, who were aware of their infection, had mild COVID-19 courses, and none of them was admitted to hospital. The overlap between seropositvity, PCR positivity and a symptomatic course are given in Fig. 3.

### Associations between potential risk factors and seroprevalence status

As shown in Table 2, there is an association between seropositivity and gender as well as some sources of infection. Employees with patient-related contact (adjusted OR 61.32, 95% CI 17.97–319.76) and professional contact (adjusted OR 7.86, 95% CI 2.38–29.17) were identified to have a larger chance of being seropositive, whereas no evidence for an association was observed between seropositive status and private contact (adjusted OR 3.66, 95% CI 0.53–25.24). While no evidence for an impact on the seroprevalence status was observed for professional groups, employees from the medium-risk area (adjusted OR 0.43, 95% CI 0.21–0.89) as well as from the low-risk area (adjusted OR 0.09, 95% CI 0.01–0.39) had a lower chance of contracting COVID-19 than employees from the high-risk area. Of note, in several multivariable models, male gender was independently from the respective risk factor (comprising several areas of private contacts and private contact as a whole, and compliance with hygienic measures) and age associated with a lower chance of being infected.

**Table 2:** Results from uni- and multivariable logistic regression modelling to investigate the association between SARS-CoV-2 seropositivity and potential risk factors

Potential risk factor	Univariable regression modelling		Multivariable regression modelling (adjusted for age and gender)					
	Potential risk factor		Potential risk factor		Adjustment: age		Adjustment: male gender	
	OR (95% CI)	*p*-value	adj. OR (95% CI)	*p*-value	adj. OR (95% CI)	*p*-value	adj. OR (95% CI)	*p*-value
Demographics								
Age, in years	0.98 (0.96, 1.01)	0.194	0.98 (0.95, 1.01)	0.140	–	–	0.37 (0.15, 0.90)	0.030
Male gender	0.39 (0.17, 0.96)	0.041	0.37 (0.15, 0.90)	0.030	0.98 (0.95, 1.01)	0.140	–	–
COVID-19 risk group (according to work area)								
High risk	ref		ref		0.99 (0.96, 1.02)	0.448	0.41 (0.17, 1.03)	0.057
Medium risk	0.43 (0.21, 0.87)	0.019	0.43 (0.21, 0.89)	0.023				
Low risk	0.08 (0.01, 0.33)	1 × 10^–4^	0.09 (0.01, 0.39)	4 × 10^–4^				
Profession^1^								
Doctor	ref		ref	ref	0.99 (0.96, 1.02)	0.565	0.57 (0.18, 1.81)	0.334
Nurses	1.38 (0.50, 4.30)	0.547	1.69 (0.53, 6.35)	0.388				
Administration	0.21 (0.02, 1.19)	0.080	0.27 (0.02, 1.69)	0.168				
Suspected source of infection								
Colleauge	19.09 (5.00, 104.46)	9 × 10^–6^	14.39 (3.69, 79.31)	8 × 10^–5^	0.99 (0.96, 1.01)	0.314	0.49 (0.19, 1.36)	0.165
Changing room	14.01 (0.73, 2060.41)	0.078	11.88 (0.60, 1772.75)	0.102	0.98 (0.95, 1.01)	0.199	0.36 (0.15, 0.89)	0.027
Recreation room	45.49 (4.70, 6077.87)	4 × 10^–4^	30.46 (3.09, 4081.37)	0.002	0.98 (0.96, 1.01)	0.240	0.45 (0.18, 1.19)	0.103
Carpool	23.96 (1.90, 3330.67)	0.013	14.80 (1.10, 2097.73)	0.042	0.98 (0.96, 1.01)	0.223	0.40 (0.17, 1.03)	0.057
Failure to maintain the minimum distance	7.94 (1.03, 88.60)	0.047	5.49 (0.69, 62.78)	0.105	0.98 (0.95, 1.01)	0.197	0.39 (0.16, 0.96)	0.042
Non-compliance with hygienic measures	4.65 (0.37, 58.34)	0.207	4.07 (0.31, 52.61)	0.253	0.98 (0.95, 1.01)	0.178	0.36 (0.15, 0.89)	0.028
Patient	74.36 (17.33, 696.59)	3 × 10^–12^	62.42 (14.53, 583.93)	4 × 10^–11^	0.99 (0.95, 1.02)	0.369	0.51 (0.18, 1.66)	0.246
Nursing activity	53.65 (12.24, 506.04)	1 × 10^–9^	47.07 (10.61, 446.23)	7 × 10^–9^	0.98 (0.95, 1.01)	0.212	0.45 (0.17, 1.35)	0.148
Highflow ventilation	69.52 (7.92, 9144.59)	9 × 10^–6^	54.05 (5.97, 7151.42)	5 × 10^–5^	0.99 (0.96, 1.02)	0.426	0.42 (0.17, 1.13)	0.082
Family/Friends	7.94 (1.03, 88.60)	0.047	6.16 (0.75, 71.13)	0.087	0.98 (0.95, 1.01)	0.170	0.39 (0.16, 0.97)	0.042
Public transport	1.51 (0.01, 28.84)	0.809	0.62 (0.00, 12.84)	0.769	0.98 (0.95, 1.01)	0.138	0.35 (0.14, 0.86)	0.023
Public events	0.90 (0.01, 11.35)	0.946	0.50 (0.00, 6.68)	0.637	0.98 (0.95, 1.01)	0.125	0.35 (0.15, 0.87)	0.024
Summary sources of infection								
Professional contact^*2^	10.49 (3.27, 38.30)	9 × 10^–5^	7.86 (2.38, 29.17)	0.001	0.98 (0.96, 1.01)	0.278	0.50 (0.20, 1.36)	0.167
Private contact^*3^	4.74 (0.71, 31.57)	0.101	3.66 (0.53, 25.24)	0.177	0.98 (0.95, 1.01)	0.179	0.38 (0.16, 0.94)	0.037
Patient related contact*^4^	73.00 (21.32, 383.19)	7 × 10^–16^	61.32 (17.97, 319.76)	2 × 10^–14^	0.99 (0.95, 1.02)	0.519	0.69 (0.22, 2.69)	0.562

For each potential risk factor, the (adjusted) odds ratio (with 95% confidence interval and *p*-value) is provided. For the multivariable models (adjusted for age and gender), the adjusted OR (with 95% CI and *p*-value) of the respective, potential risk factor as well as of the variables, the model was adjusted for, are provided. The models were estimated based on 224 participants

*CI* confidence interval, *MTA* medical technical assistant, *N* number of; (adj.) *OR* (adjusted) odds ratio, *ref.* reference, *RTA* radiological technical assistant

^*1^139 people in the model, partly because various professions were excluded due to low representation

^*2^Colleagues, changing room, recreation room, carpool, failure to maintain the minimum distance

^*3^Family/Friends, public transport, public events

^*4^Patient, nursing activities, highflow ventilation

### Compliance during aerosol generating measures

**Fig. 4:**
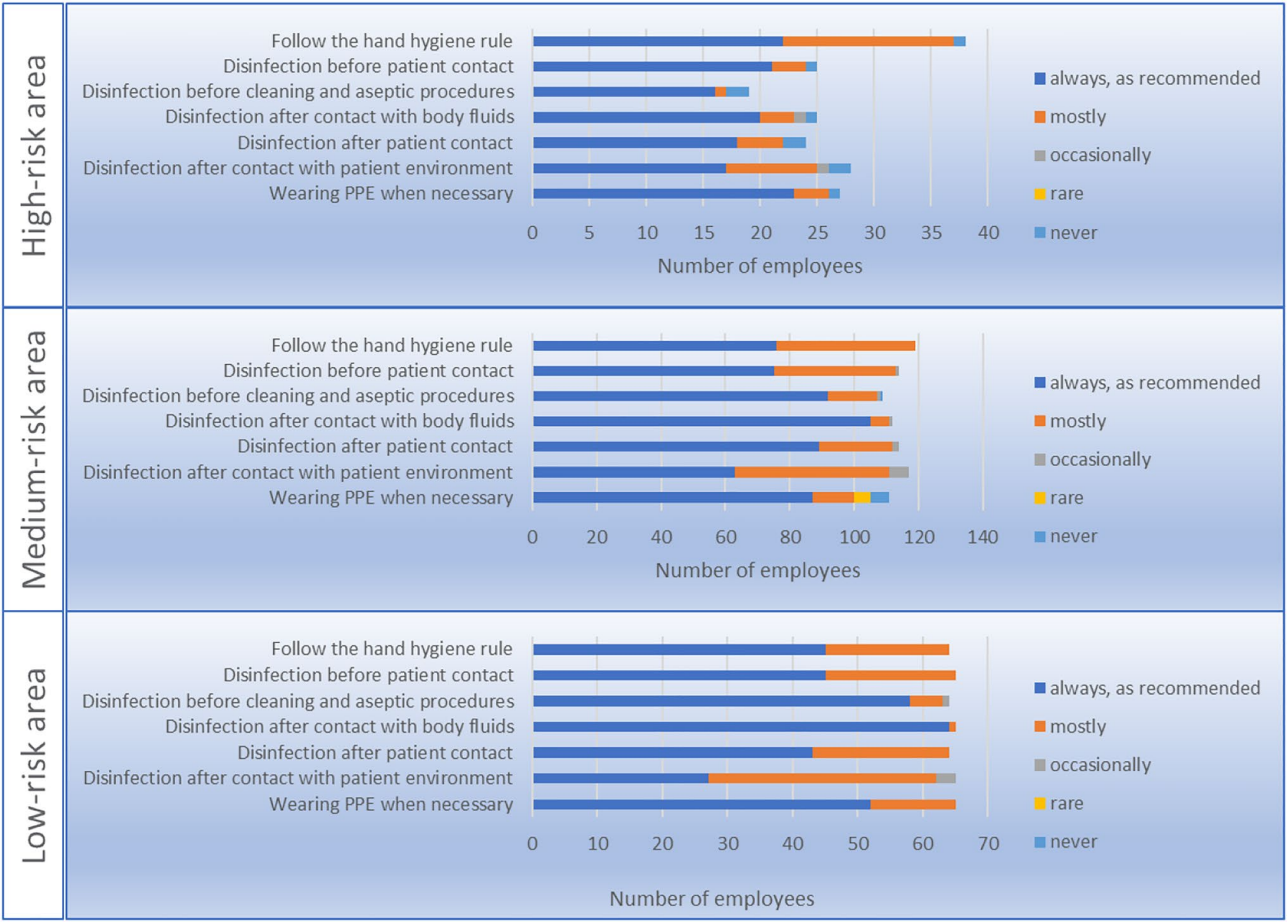
Compliance with recommended hygiene measures and wearing of personal protective equipment (PPE) when in contact with COVID-19 patients. *PPE* personal protective equipment

Thirty-five (15.3%) of the 229 hospital employees reported presence on the ward during aerosol-generating measures related to COVID-19 patients. Ten of them reported that room doors were not closed during these measures. Of these 35 employees, 13 were seropositive. An overview on compliance on general hygiene measures is provided in Fig. 4.

## Discussion

The main findings of our study are as follows: Overall, the rate of SARS-CoV-2 infected staff members based on PCR and serostatus was with 17.5% higher than the seroprevalence rate of 2.7% observed in a study at Jena University Hospital (JUH) that was conducted at the same time in the same region [2]. In contrast to the study at the JUH, where private contact, but not patient-related contact was identified as the main risk factor for a positive COVID-19 serostatus (reflected by the highest seroprevalence in the low-risk group and the lowest in the high-risk-group), the results in our study were vice versa. The seroprevalence rate was highest in the high-risk group, while it was lowest in the low-risk group.

Noteworthy, exposure to a high-flow ventilated patient via open doors and without active negative pressure in the patient room was identified as associated with a positive serostatus despite wearing a surgical mask by the health-care workers becoming infected. This observation, despite the wide confidence intervals, underlines the WHO recommendation to use FFP2/KN95 masks in proximity to aerosol generating procedures [3]. In the hospital, in which the study was conducted, the outbreak could be terminated, and further outbreaks prevented by improving indoor air conditions, as well as introducing a continuing masking requirement using FFP2, initially in aerosol-generating measures, and subsequently throughout the hospital. After the outbreak, no further nosocomial human-to-human (employee/patient) transmission was detected despite increased awareness. The source of infection for patients 2 and 3 could not be clearly identified.

This outbreak report points toward a significant increased risk of spread of the infection within the staff by carpooling and the common use of the recreation room, both frequently without masking, which is underlined by a Japanese study from 2020 [17]. This underlines that pandemic preparedness of hospitals should include splitting teams into fixed groups that do not mix—ideally even not outside the hospital—to reduce the risk of transmission within the staff.

Numerous previous reports suggested that male gender is a risk factor for severe COVID-19 infections [6, 16]. A different unexpected gender-specific aspect emerged from our multivariable analyses: females had at higher chance of infection despite being exposed to the same potential risk factors as their male colleagues. This gender-specific increased risk of SARS-CoV-2 infection may be linked to differences in social interaction patterns. Recent research employing sociometers has shown that women tend to be more physically proximate and communicative with one another, particularly in collaborative settings, compared to men [13].

Strengths of the paper include that the facility is a small hospital and therefore all employees could be reached. Furthermore, the outbreak occurred early in the pandemic, so the outcome was not skewed by the introduction of vaccination or high-incidence rates in the private setting of the employees. In addition, asymptomatic infections were also detected in our study, in which the seroprevalence was determined with more than one test.

A general limitation of outbreak observations is that they are not randomized trials. In our study, additional exposure to other sources was reported, particularly to infected colleagues, or different inter-individual and intra-individual compliance with wearing PPE. Besides the general methodological limitations of an outbreak report, the following limitations have to be taken into account: the questionnaire was not distributed until 189 days after the outbreak, not all staff participated in the study, and different hygiene measures were implemented at different time points during the outbreak. Data on adherence to hygienic measure and possible expositions were collected 26 weeks after the outbreak. Therefore, the responses in the questionnaire might be less accurate compared to an assessment during the outbreak. In summary, our analyses might be susceptible to selection bias (related to the study population) as well as both recall bias and reporting bias (related to answering the questionnaire). The identified associations of seropositivity with potential risk factors might be biased by not realized or not reported exposures to SARS-CoV-2 outside the working environment/place.

In conclusion, our study underscores the critical importance of using FFP2/KN95 masks in close proximity to patients with high-flow ventilation and highlights the need for vigilant infection control measures within healthcare facilities. Particularly, the risk of within-staff transmission through common areas and carpooling must not be underestimated. The early implementation of strict team segregation protocols when facing easily transmissible respiratory infections with short incubation periods, such as COVID-19, is essential to mitigate the risk of outbreaks within healthcare settings [11, 12].

## Data Availability

The data are available and can be requested from the authors for further analysis subject given a renewed positive opinion from the ethics committee and the data protection authority for the intended further analyses.
